# Supplementary material for: Coccolith clumped isotopes reveal modest rather than extreme northern high latitude amplification during the Miocene
Source: Nat Commun. 2025 Dec 9;16:10981. doi: 10.1038/s41467-025-65954-y (PMC12689775; doi:10.1038/s41467-025-65954-y)
Supplement: Supplementary file 1 — Supplementary Information [file 41467_2025_65954_MOESM1_ESM.pdf]

# Supplementary information for

## Coccolith clumped isotopes reveal modest rather than extreme northern high latitude amplification during the Miocene

Luz María Mejía[1,2*], Stefano M. Bernasconi[1], Alvaro Fernandez[3], Hongrui Zhang[1,4], José Guitián[1,5], Madalina Jaggi[1], Victoria E. Taylor[6], Alberto Perez-Huerta[7], Heather Stoll[1]

[1] Geological Institute, ETH Zürich, Sonneggstrasse 5, ETH, 8092, Zürich, Switzerland
[2] Now at MARUM, University of Bremen, 28359 Bremen, Germany
[3] Instituto Andaluz de Ciencias de la Tierra, Av. de las Palmeras 4, 18100 Armilla, Granada, Spain
[4] Now at Tongji University, Siping Road 1239, Shanghai, China
[5] Now at Department of Oceanography, Instituto de Investigacións Mariñas, Consejo Superior de Investigaciones Científicas (CSIC), Vigo, Spain
[6] Department of Earth Science and Bjerknes Centre for Climate Research, University of Bergen, Allègaten 41, 5007, Bergen, Norway
[7] Department of Geological Sciences, University of Alabama, Tuscaloosa, AL 35487, USA

* Corresponding author email: lmejia@marum.de

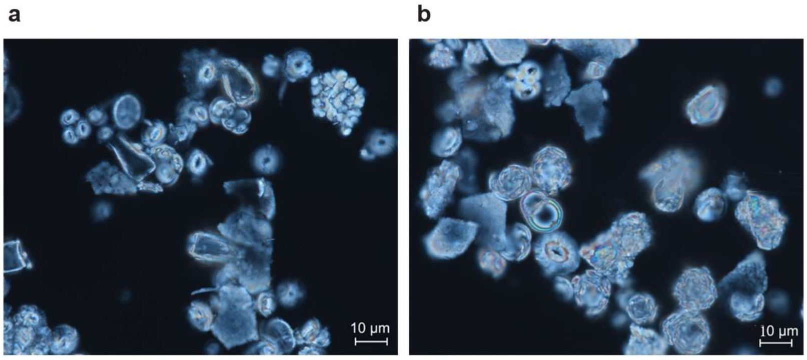


**Supplementary Figure 1. Example of light microscope image of the 10-11 µm size fractions**. **a** 4.17 Ma. **b** 5.79 Ma. This fraction is enriched in large non-coccolith fragments like foraminifera fragments.

### Supplementary Note 1. Negligible cold bias effect of *Coccolithus pelagicus* in coccolith $\Delta_{47}$ temperatures

In addition to reticulofenestrids, other species in the assemblages include *C. pelagicus*, *Calcidiscus* sp., *Helicosphaera* sp., *Sphenolithus* sp., *Discoaster* sp., and *Postosphaera* sp. (Supplementary Fig. 2). Sediment trap studies from the nearby North Atlantic Bloom Experiment 48 (NABE) have shown that increased abundances of *C. pelagicus* are not observed when all other coccolithophore species have a blooming peak during spring [1]. *C. pelagicus* is a dominant species in subpolar North Atlantic waters with an optimum temperature range of 2-12 °C [2,3] and its increased abundance has been related to the presence of cyclonic eddies in the area, most likely transporting them from higher to lower latitudes [1,4]. Therefore, they are unlikely to represent a large part of the *in situ* coccolithophore production in our ODP Site 982. The presence of subpolar eddies in the area has been suggested to lead to cold biases in alkenone temperatures [4,5]. Therefore, the increased relative abundance of *C. pelagicus* in our samples at ~2, 4.2 and 14 Ma (4.2, 3.4, and 10.6%, respectively) could potentially result in a cold bias in our clumped isotope temperatures as well, possibly related to a more frequent influence of subpolar eddies.

From these three samples, however, only at ~14 Ma are clumped isotope temperatures for the large size fraction (8-10 µm; 11.02 ± 3.9 °C, 95% CI) significantly colder than for the average of other size fractions (3-5 and 5-8 µm; 21.58 ± 3.33 °C, 95% CI), and is the relative abundance of *C. pelagicus* high enough to produce a cold bias. Applying simple mass balance, and assuming all coccoliths in the 8-10 µm size fraction are *C. pelagicus* advected from colder latitudes (which is not the case), the temperature underestimation of this sample would remain 1.1 °C, which is smaller than the analytical error of clumped isotope measurements. Therefore, we can conclude that the presence of *C. pelagicus* cannot explain the observed temperature differences between alkenone and clumped isotope temperatures.

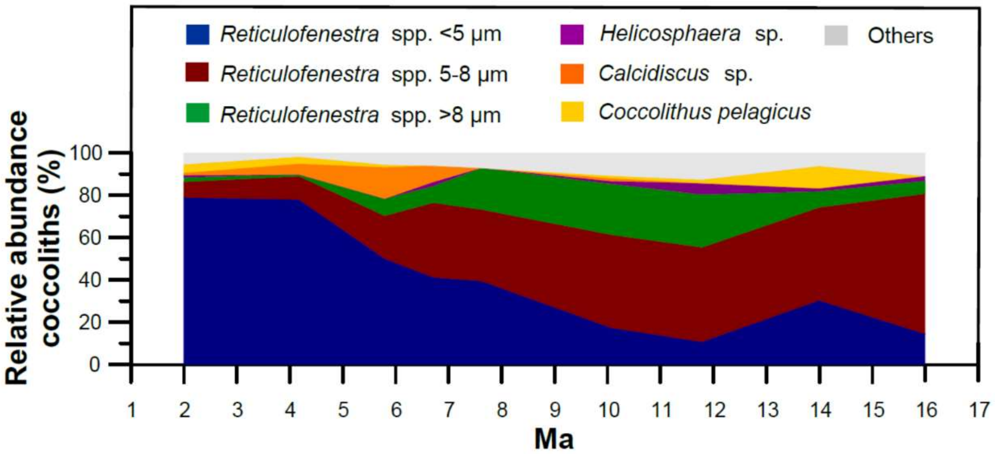


**Supplementary Figure 2. Relative coccolith abundances (%) in the assemblages of sediments from ODP Site 982.** Coccolith counting was estimated from the 2-10 µm. Dominant coccolithophore species are *Reticulofenestra* spp. <5 µm, *Reticulofenestra* spp. 5-8 µm, *Reticulofenestra* spp. >8 µm, *Helicosphaera* sp., *Calcidiscus* sp., and *Coccolithus pelagicus*. Other species include *Sphenolithus* sp., *Discoaster* sp., and *Postosphaera* sp., and are grouped together with the unidentifiable >2 µm carbonate fragments as "others".

**Supplementary Note 2. Negligible cold biases in coccolith $\Delta_{47}$ temperatures from diagenetic processes**

Clumped isotope thermometry is sensitive to the presence of diagenetically-altered carbonate [6]. In the case of coccolith calcite, carbonate overgrowth at the seafloor occur at colder temperatures than primary biological calcification in the euphotic ocean. Therefore, diagenesis can potentially bias reconstructed temperatures towards colder values. For our high latitude ODP 982 Site, where the temperature gradient with water depth is smaller compared to warm and more stratified waters in the tropics, diagenetic alteration is expected have a lower impact in $\Delta_{47}$ reconstructed temperatures.

SEM shows the generally good coccolith preservation in all samples (Supplementary Fig. 3). Yet, regardless of the burial time, there is some carbonate overgrowth partially or completely covering the central area (Supplementary Fig. 4), but in coccoliths of all ages, the rims are well defined. Overall, authigenic carbonate comprises a low proportion of analyzed carbonate. Our upper estimate of authigenic carbonate is always < 8.1% (5.8 Ma sample) and in some cases as low as 2.8% (11.8 Ma sample) (Table S1).

To understand the potential effect of post-burial alteration on $\Delta_{47}$ temperatures, we applied the diagenesis model of Stolper et al. [7] to ODP Site 982. This model estimates quantitatively the effect of diagenesis on $\Delta_{47}$ temperatures. To apply the model, we used the recrystallization rates estimated by Schrag et al. [8] for bulk carbonates in the equatorial ODP Site 807, bottom water temperatures from Lear

et al. [9], average sedimentation rates of our ODP Site 982 core (36.1 meters per million years), a geothermal gradient of 30 °C per km of sediment buried, and for initial temperatures (before alteration), the alkenone derived SSTs. The results indicate larger cold offsets in older samples, with a trend of increasing diagenetic alteration (%) over time (Supplementary Fig. 5a). The oldest sample (16 Ma) show up to 55% diagenetic alteration, and the youngest sample (2 Ma) > 10%. These amounts are much larger than the upper estimates of authigenic carbonate in our samples, and they are the direct result of using recrystallization rates for bulk carbonates rather than for coccolith calcite. Since coccoliths are covered with a protective polysaccharide organic matrix which makes them resistant to carbonate alteration [10], it is expected that the high recrystallization rates observed by Schrag et al. [8] result mainly from non-coccolith carbonate. Thus, the model overestimates the effects of authigenic contributions in our samples since recrystallization rates specific to the pure coccolith fractions are expected to be significantly lower.

Although actual coccolith recrystallization rates are unknown, the model can be adjusted with lower recrystallization rates, thus allowing it to predict diagenetic alteration consistent with our overgrowth observations (e.g., < 8.1%). This was done using fractional amounts of the bulk carbonate recrystallization rates from 0.1 to 0.25 times (in increments of 0.01). Results show that the cold bias expected for the amounts of authigenic carbonate we observe is always < 2°C (Supplementary Fig. 5b). Finally, as a third way to estimate the potential alteration effect, we calculate it using a simple mass balance model. We use the alkenone SST as initial temperature and deep-water temperatures (diagenesis temperature) as endmembers, and the maximum amount of overgrowth that we observe as the fraction of diagenetic calcite in each sample (f=0.081). The results are very similar to the results of the modified diagenesis model; expected offsets in $\Delta_{47}$ temperatures relative to the alkenone data are ~2°C (Supplementary Fig. 5c).

Although some dissolution was observed in coccoliths of all samples, particularly in the smallest and thinnest, to date, there is no evidence that dissolution can affect clumped isotope-derived temperatures. Moreover, compared to other marine organisms like foraminifera, whose calcite is composed by several nanometer-sized crystals [11], a coccolith is a single calcite crystal characterized by its homogeneous chemistry composition (e.g. ref. [12]). In addition to being restricted to the relatively thermally-stable photic zone and not showing vertical migration behavior like foraminifera, coccolithophores can produce single coccoliths intracellularly within one hour [13], highly restricting the possibility of clumped isotopes of a single coccolith to register different temperatures during its formation. Therefore, it is highly unlikely that removal of calcite from etching can affect clumped isotope-derived temperatures from coccoliths.

Smaller size fractions, especially the fragmented ones (like the <2 µm) are expected to be more prone to diagenetic alteration compared to whole coccoliths. This is not only because the surface area to volume is higher and therefore there is more surface of interaction with water, but also because the protective polysaccharides [10,14] may have been removed from coccolith fragments. The lack of this organic protective cover could also increase the probability of diagenetic processes affecting fragments compared to whole coccoliths. Therefore, we would expect size fractions containing important amounts of <2 µm fragments (i.e. <11 µm size fraction) to be more affected by diagenesis. However, the similar clumped isotope-derived temperatures of the <11 µm size fractions and the pure coccolith (2-10 µm) size fractions (Fig. 2), which are <2 µm free, suggest that at Site 982 most of the <2 µm fragments are composed of relatively well-preserved coccolith fragments. Moreover, the Sr/Ca ratios of the <2 µm fractions (1.40-1.88 mmol/mol) are typical for coccoliths found in cultures, sediment traps and sediment cores [15], are similar to those shown by pure coccolith size fractions in this study (2-10 µm: 1.69-2.02 mmol/mol), and are higher than expected for abiogenic calcite precipitated from seawater or pore fluids [16] (Table S2). This suggests that the Mg and Al enrichment shown by trace element analysis in the <2 µm fraction is not mainly driven by diagenetic processes, but rather by an enrichment of clay. The presence of clay minerals around small coccolith fragments could have contributed to a better preservation of this size fraction.

The very low authigenic carbonate from our samples shows that the removal of <2 µm fragments from the pure coccolith 2-10 µm size fraction would have not been necessary. However, the removal of this diagenetically susceptible fraction may be required in other sediments where this fraction is altered, even if its removal increases separation time in at least ten times. This applies to old sediments, in which diagenesis is expected to have had more time to affect pristine carbonate, but also to recent ones in locations where detrital sediments in the small size fraction are important [17]. Since in tropical, warm, stratified locations and time intervals temperature differences between surface and bottom waters are larger than in high latitudes like ODP Site 982, a special evaluation of the diagenetic component of these sediments is required to ensure accurate temperature reconstructions using coccolith clumped isotopes.

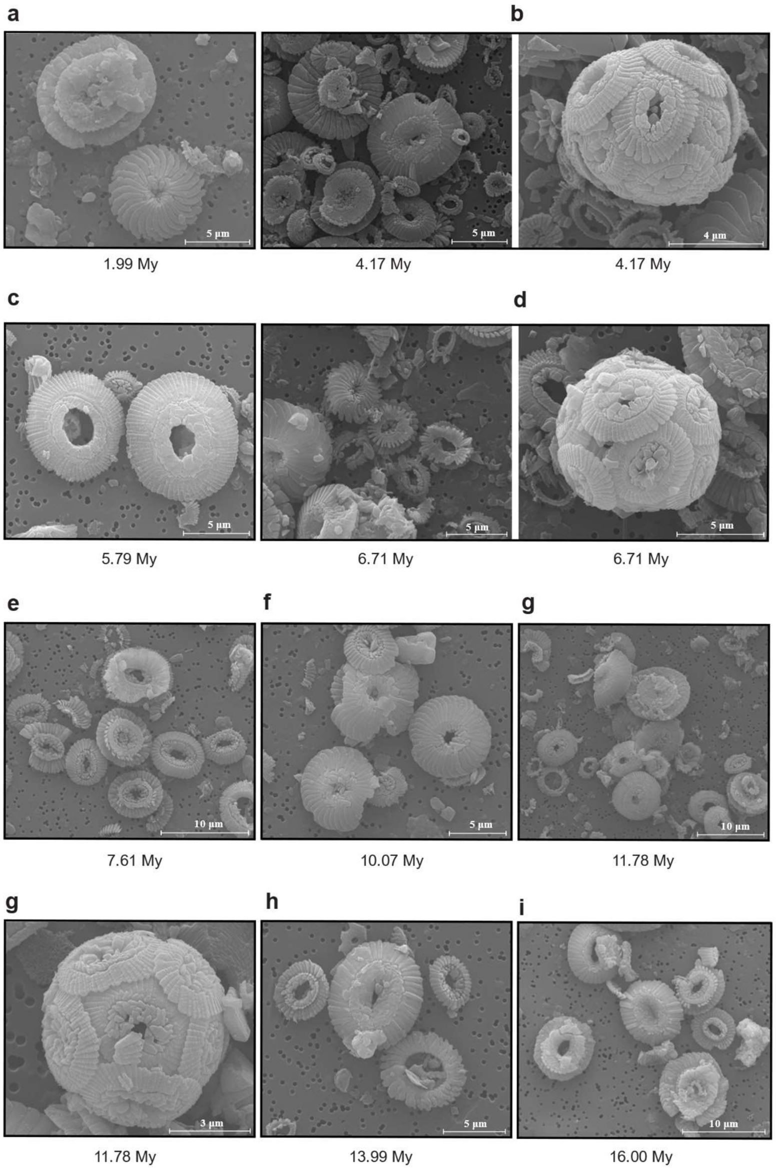


**Supplementary Figure 3. Scanning Electron Microscope images of < 11 µm coccolith fractions from ODP Site 982. a** 1.99 Ma. **b** 4.17 Ma. **c** 5.79 Ma. **d** 6.71 Ma. **e** 7.61 Ma. **f** 10.07 Ma. **g** 11.78 Ma. **h** 13.99 Ma. **i** 16 Ma. Note that the < 11 µm size fraction contains the <2 µm and therefore some small carbonate and clay fragments are deposited on top of coccoliths and coccospheres.

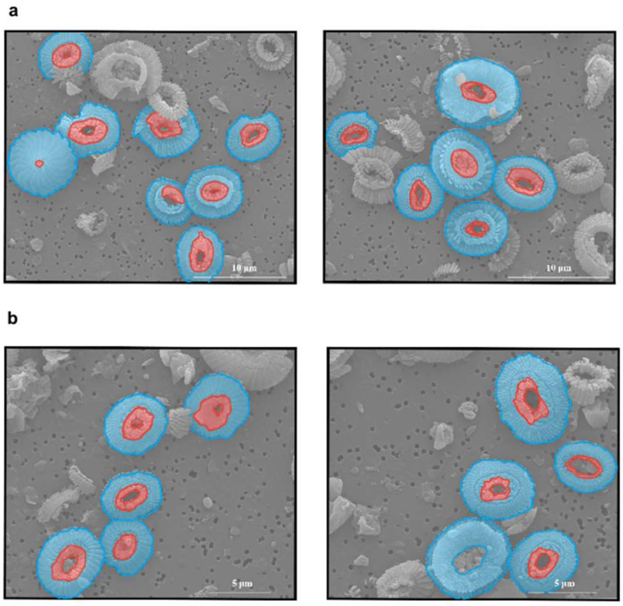

**Supplementary Figure 4. Example of scanning Electron Microscope images of <11 µm coccolith fractions used for estimating coccolith surface area affected by diagenesis. a** 7.61 Ma. **b** 10.07 Ma. Areas shaded in blue and red denote areas with well preserved and affected calcite, respectively. Note that the < 11 µm size fraction contains the <2 µm and therefore some small carbonate and clay fragments are deposited on top of coccoliths, but may not be authigenic calcite.

**Supplementary Table 1. Pristine and diagenetically altered carbonate in samples.** Area of pristine and diagenetically altered carbonate (%) from SEM imaging, and amount of pristine and diagenetically altered carbonate (%), calculated following the geometrically-calculated coccolith volume plots of Young and Ziveri [18], and assuming that maximum half of the calculated volume was affected by diagenesis.

| Age (Ma) | % area SEM images | | % calcite in samples | |
|---|---|---|---|---|
| | **Pristine coccolith** | **Authigenic calcite** | **Pristine coccolith** | **Authigenic calcite** |
| 1.99 | 83.9 | 16.1 | 93.1 | 6.9 |
| 4.17 | 89.0 | 11.0 | 96.5 | 3.5 |
| 5.79 | 82.1 | 17.9 | 91.9 | 8.1 |
| 6.71 | 88.5 | 11.5 | 95.9 | 4.1 |
| 7.61 | 85.9 | 14.1 | 94.3 | 5.7 |
| 10.07 | 89.2 | 10.8 | 96.5 | 3.5 |
| 11.78 | 90.4 | 9.6 | 97.2 | 2.8 |
| 13.99 | 83.8 | 16.2 | 93.1 | 6.9 |
| 16 | 85.7 | 14.3 | 94.3 | 5.7 |

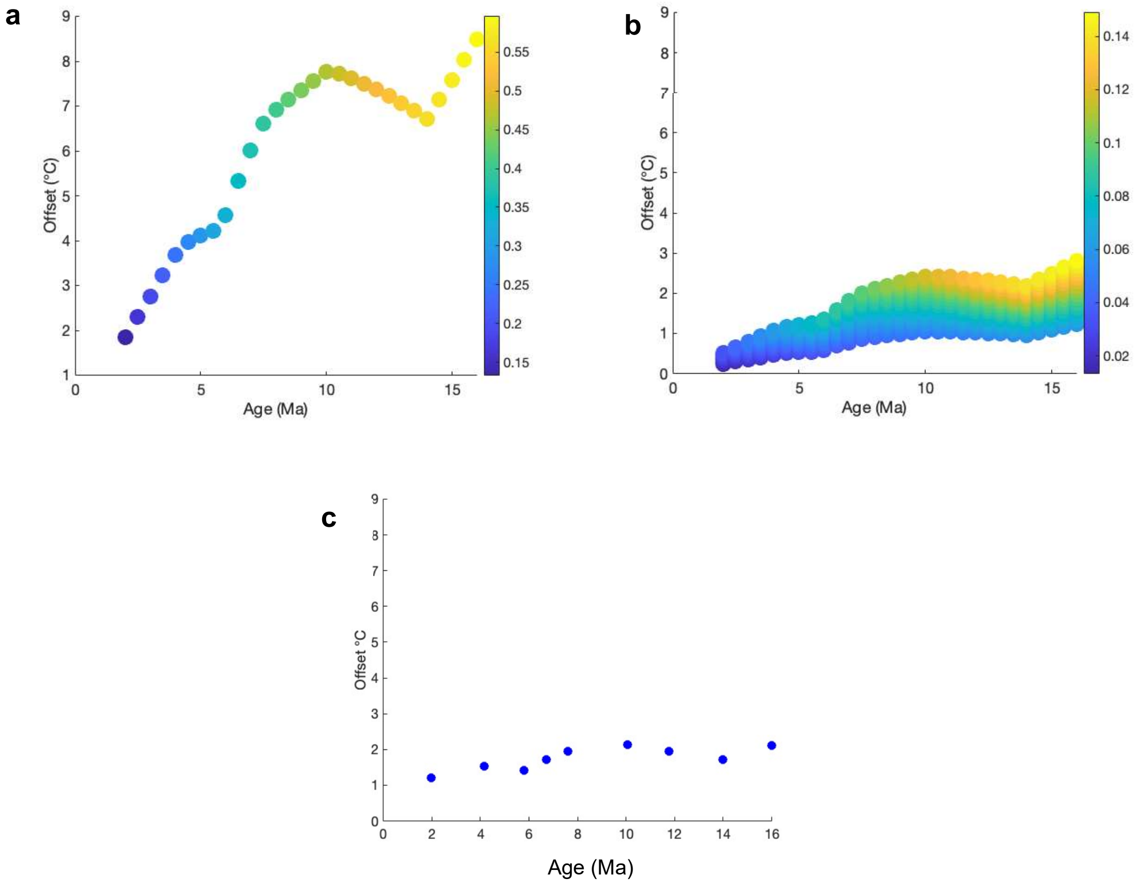


**Supplementary Figure 5**. **Effect of recrystallization on coccolith clumped isotope temperatures. a** Offsets between alkenone SSTs and coccolith $\Delta_{47}$ temperatures estimated with the diagenesis model of Stolper et al. [7] and the recrystallization rates of Schrag et al. [8] for bulk carbonate. **b** Offsets between alkenone SSTs and coccolith $\Delta_{47}$ temperatures estimated with the diagenesis model of Stolper et al. [7] and with fractional amounts (10-25%) of the recrystallization rates of Schrag et al. [8] for bulk carbonate. **c** Offsets between alkenone SSTs and coccolith $\Delta_{47}$ temperatures estimated with a mass balance model. Colorbar in A and B is fraction carbonate recrystallized.

**Supplementary Table 2. Trace element analysis of the < 2 and the 2-10 µm size fractions from ODP Site 982.**

| Age (Ma) | Size fraction (µm) | Sr/Ca (mmol/mol) | Mg/Ca (mmol/mol) | Al/Ca (mmol/mol) |
|---|---|---|---|---|
| 1.99 | | 1.84 | 4.54 | 3.01 |
| 4.17 | | 1.46 | 11.35 | 2.83 |
| 5.79 | | 1.40 | 5.69 | 1.70 |
| 6.71 | | 1.57 | 4.84 | 1.97 |
| 7.61 | <2 | 1.61 | 8.30 | 3.50 |
| 10.07 | | 1.54 | 5.75 | 1.35 |
| 11.78 | | 1.55 | 4.49 | 1.46 |
| 13.99 | | 1.53 | 3.11 | 0.90 |
| 16.00 | | 1.88 | 4.03 | 1.44 |
| 1.99 | | 2.01 | 2.44 | 0.67 |
| 4.17 | | 2.02 | 2.10 | 0.07 |
| 5.79 | | 1.74 | 1.97 | 0.12 |
| 6.71 | | 1.76 | 1.18 | 0.11 |
| 7.61 | 2-10 | 1.78 | 1.14 | 0.17 |
| 10.07 | | 1.69 | 1.96 | 0.28 |
| 11.78 | | 1.79 | 1.95 | 0.03 |
| 13.99 | | 1.79 | 1.70 | 0.17 |
| 16.00 | | 1.90 | 1.51 | 0.34 |

## Supplementary Note 3. Differences in calibration approaches: "Depth of production effect"

For the North Atlantic, significantly high depth-integrated phytoplankton biomasses and chlorophyll inventories have been observed using floats [19] and by satellite and modelling studies during the cold period of mixed layer deepening (December-February) [20]. A deeper production could contribute to temperature differences between coccolith clumped isotope and alkenone proxies. This "depth of production effect" is expected to be larger in lower latitudes like in the oligotrophic South Pacific and North Pacific gyres, where peak production at depth (150-200 [21] and 75-100 m [22], respectively) has been described. The same is valid for warmer intervals, as more stratified waters are expected to increase this "depth of production effect", wherewith differences between absolute reconstructions using widely-used alkenone calibrations [23,24] (SSTs) vs. coccolith clumped isotopes (temperatures at depth of production) are also expected to be larger.

To estimate the magnitude of the "depth of production effect" for the modern North Atlantic, we calculated the differences of World Ocean Atlas (WOA) 2018 [25] average monthly temperatures between surface waters and those at 40 and 100 m for months when integrated depth and surface primary production is expected to be significant (i.e. ~from December to May/June [20]; Table S3). Depths between 40 and 100 m were chosen, as 1984 cruise data for our study site from April, which is one of the months with both the highest "surface" phytoplankton biomass and coccolithophore fluxes [1,19,20] show significantly larger chlorophyll values between 40 and 100 m, with a peak at 60 m [26]. A maximum temperature difference between surface and deeper waters of 1.6 °C was observed for June, assuming peak of production at 100 m, with decreasing magnitudes for earlier months, when the mixed layer is deeper.

**Supplementary Table 3. Average monthly temperatures from WOA between 1955 and 2012 for the surface ocean (0 m), at 40 and 100 m depth, for the location of ODP Site 982.** Average temperatures at the same depths for the winter-spring production season [20], and differences of average monthly temperatures between surface (0 m) and 40 m and surface and 100 m depth, also shown. These differences show that for months when integrated depth and surface primary production is expected to be significant (~from December to May/June; shown in italics), alkenone temperatures calculated using SST could be up to 1.6 °C (italics, bold) higher than deeper temperatures at which alkenones may be actually produced.

| | **Temp. (°C); 0 m** | **Temp. (°C); 40 m** | **Temp. (°C); 100 m** | **Temp. diff. (°C); 0-40 m** | **Temp. diff. (°C); 0-100 m** |
|---|---|---|---|---|---|
| ***Jan*** | *9.44* | *9.40* | *9.37* | *0.04* | *0.07* |
| ***Feb*** | *9.03* | *9.04* | *8.99* | *-0.01* | *0.04* |
| ***Mar*** | *8.95* | *8.89* | *8.91* | *0.06* | *0.04* |
| ***Apr*** | *9.21* | *9.02* | *8.93* | *0.19* | *0.28* |
| ***May*** | *9.96* | *9.52* | *9.18* | *0.44* | *0.78* |
| ***Jun*** | *10.84* | *10.16* | *9.24* | *0.68* | ***1.60*** |
| **Jul** | 12.47 | 10.95 | 9.34 | 1.52 | 3.13 |
| **Aug** | 13.20 | 11.69 | 9.51 | 1.51 | 3.69 |
| **Sep** | 12.51 | 12.06 | 9.75 | 0.45 | 2.76 |
| **Oct** | 11.47 | 11.33 | 10.09 | 0.14 | 1.38 |
| **Nov** | 10.20 | 10.11 | 10.00 | 0.09 | 0.20 |
| ***Dec*** | *9.51* | *9.49* | *9.45* | *0.02* | *0.06* |
| **Av. winter-spring** | *9.60* | *9.40* | *9.20* | 0.22 | *0.45* |

**Supplementary Note 4. Differences in calibration approaches: "Season of production effect"**

In places where alkenone production is seasonal, like in the North Atlantic, coretop calibrations using annual [23] or warm season [24] SSTs, may introduce seasonal biases in $U^{k'}_{37}$ temperatures [27]. Therefore, the ideal calibration should use temperatures of periods when most of the alkenones that are preserved in the sediment are produced. We calculated the temperature differences potentially caused by this "season of production effect". For this, we compared WOA average monthly SSTs between the months used by alkenone calibrations (all year [23], August-October [24]) and those of months reported to coincide with maximum surface production in the North Atlantic [1,4,19,20,28], or significant depth-integrated primary production [20] (winter-spring; Table S4). Flux peaks in sediment traps may lag maximum surface chlorophyll by 1-2 months due to long settling times [29]. The maximum coccolith export in March-May recorded by the 1 km trap at the nearby NABE-48 site [1] and the slightly later alkenone flux peak in the deeper 3.7 km trap (April-June [4]) are also consistent with winter-spring production [20]. This simple analysis shows that the application of the BAYSPLINE calibration [24], which uses significantly warmer temperatures than those of actual alkenone production, can lead to up to 3°C overestimates in alkenone-calculated temperatures. Smaller overestimates are estimated when the core top calibration [23] is used (up to 1.2 °C).

**Supplementary Table 4. Average monthly SSTs from WOA between 1955 and 2012 for the location of ODP Site 982 in the North Atlantic.** This includes average monthly SSTs used for the alkenone BAYSPLINE [24] and the core top [23] calibrations; average monthly SSTs of periods of surface coccolith peak export [1], alkenone peak export [4], and phytoplankton surface blooms [19,20,28] in the North Atlantic, and average monthly SSTs of periods of significant depth-integrated and surface phytoplankton production in the North Atlantic [20]. Temperature differences between average monthly SSTs of considered periods for alkenone calibrations and actual production periods, show that the maximum "season of production effect" can reach up to 3.0 °C when comparing the BAYSPLINE calibration and the Broerse et al. [1] dataset (bold, italics).

| | | **Surface** | | | | | | | **Depth-integrated + surface** |
|---|---|---|---|---|---|---|---|---|---|
| **WOA average monthly SST (°C)** | | **BAYSPLINE [24] Aug-Oct** | **Core top [23] Mean annual** | **Filippova et al. [28] Mar-Aug** | **Mignot et al. [19] Apr-May** | **Broerse et al. [1] Mar-May** | **Rosell-Melè et al. [4] Apr-Aug** | **Behrenfeld et al. [20] Apr-Jul** | **Behrenfeld et al. [20] Dec-Jun** |
| **Jan** | **9.44** | | | | | | | | |
| **Feb** | **9.03** | | | | | | | | |
| **Mar** | **8.95** | | | | | | | | 9.6 |
| **Apr** | **9.21** | | | | 9.6 | 9.4 | | | |
| **May** | **9.96** | | | 10.8 | | | | 10.6 | |
| **Jun** | **10.84** | | 10.6 | | | | 11.1 | | |
| **Jul** | **12.47** | | | | | | | | |
| **Aug** | **13.20** | | | | | | | | |
| **Sep** | **12.51** | 12.4 | | | | | | | |
| **Oct** | **11.47** | | | | | | | | |
| **Nov** | **10.20** | | | | | | | | |
| **Dec** | **9.51** | | | | | | | | |
| **Difference to BAYSPLINE** | | | | 1.6 | 2.8 | ***3.0*** | 1.3 | 1.8 | 2.8 |
| **Difference to core top** | | | | -0.2 | 1.0 | 1.2 | -0.6 | -0.1 | 1.0 |

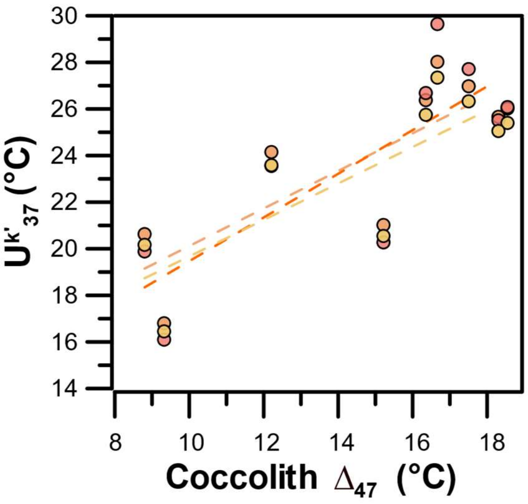


**Supplementary Figure 6. Alkenone SSTs as a function of coccolith clumped isotope temperatures from ODP Site 982 samples**. Positive correlations between proxies are significant and are calculated using temperatures from the 2-10 µm coccolith size fractions. Correlations obtained using the *E. huxleyi* 55a batch culture [30] ($r = 0.82$, $p = 0.007$), the core top [23] ($r = 0.82$, $p = 0.007$) and the BAYSPLINE [24] ($r = 0.80$, $p = 0.009$) calibrations shown in light blue, green and orange, respectively.

**Supplementary Table 5. Maximum and minimum temperatures derived from applying eleven different *Emiliania huxleyi* and *Gephyrocapsa oceanica* batch culture calibrations [31] to our ODP Site 982 $U^{k'}_{37}$ measurements.** This includes the widely used *E. huxleyi* 55a batch culture calibration of Prahl et al. [30]. Temperature differences between culture calibrations can reach up to 8 °C for a given $U^{k'}_{37}$ value (bold, italics). Alkenone temperatures obtained using the BAYSPLINE [24] and the core top [23] calibrations, and coccolith clumped isotope temperatures are shown for comparison.

| **Age (Ma)** | $U^{k'}_{37}$ | **$U^{k'}_{37}$ culture calibr.[30,31] (°C)** | | **Max-Min culture calibr.[30,31] (°C)** | **BAYSPLINE [24] $U^{k'}_{37}$ (°C)** | **Core top[23] $U^{k'}_{37}$ (°C)** | **Coccolith $\Delta_{47}$ (°C)** |
|---|---|---|---|---|---|---|---|
| | | **Max** | **Min** | | | | |
| 1.99 | 0.5984 | 23.8 | 16.5 | 7.4 | 16.1 | 16.8 | 9.3 |
| 4.17 | 0.7377 | 27.7 | 20.5 | 7.2 | 20.3 | 21.0 | 15.2 |
| 5.79 | 0.7247 | 27.4 | 20.2 | 7.2 | 19.9 | 20.6 | 8.8 |
| 6.71 | 0.8410 | 30.6 | 23.4 | 7.2 | 23.6 | 24.2 | 12.2 |
| 7.61 | 0.9026 | 32.3 | 24.9 | 7.4 | 26.1 | 26.0 | 18.5 |
| 10.07 | 0.9342 | 33.2 | 25.5 | 7.7 | 27.7 | 27.0 | 17.5 |
| 11.78 | 0.9145 | 32.7 | 25.2 | 7.5 | 26.7 | 26.4 | 16.4 |
| 13.99 | 0.8908 | 32.0 | 24.7 | 7.4 | 25.5 | 25.7 | 18.3 |
| 16.00 | 0.9687 | 34.2 | 26.2 | ***8.0*** | 29.6 | 28.0 | 16.7 |

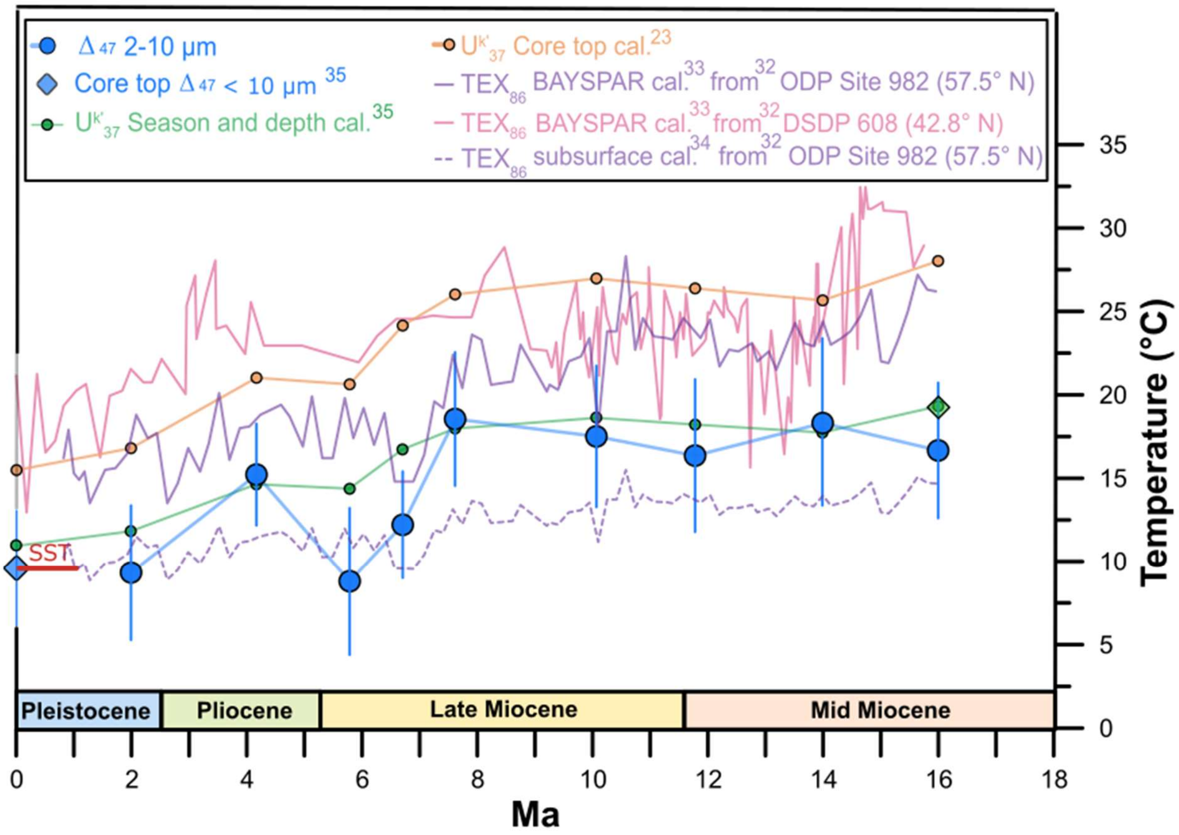


**Supplementary Figure 7. Coccolith clumped isotope, alkenone, and $TEX_{86}$ temperature evolution in ODP Site 982 and DSDP Site 608 (subtropical gyre).** $TEX_{86}$ temperatures from DSDP Site 608 (pink line) and from ODP Site 982 (purple line) from the study of Super et al.[32] using the BAYSPAR calibration[33], showing similar absolute values despite the 14.7° difference in latitudes. We also include ODP Site 982 $TEX_{86}$ temperatures calculated using the subsurface calibration [34] (dashed purple), which show absolute magnitudes generally lower than coccolith $\Delta_{47}$ calcification temperatures. We include temperatures from ODP Site 982 (this study) derived from alkenones applying the core top [23] (orange dots) and a calibration that considers the season and depth of production [35] (green dots), and coccolith $\Delta_{47}$ calcification temperatures (2-11 μm: blue dots). Coretop alkenone and coccolith $\Delta_{47}$ temperatures from the study of Mejía et al. [35] in our same Site are also included. Error bars in coccolith $\Delta_{47}$ calcification temperatures record denote the 95% CI.

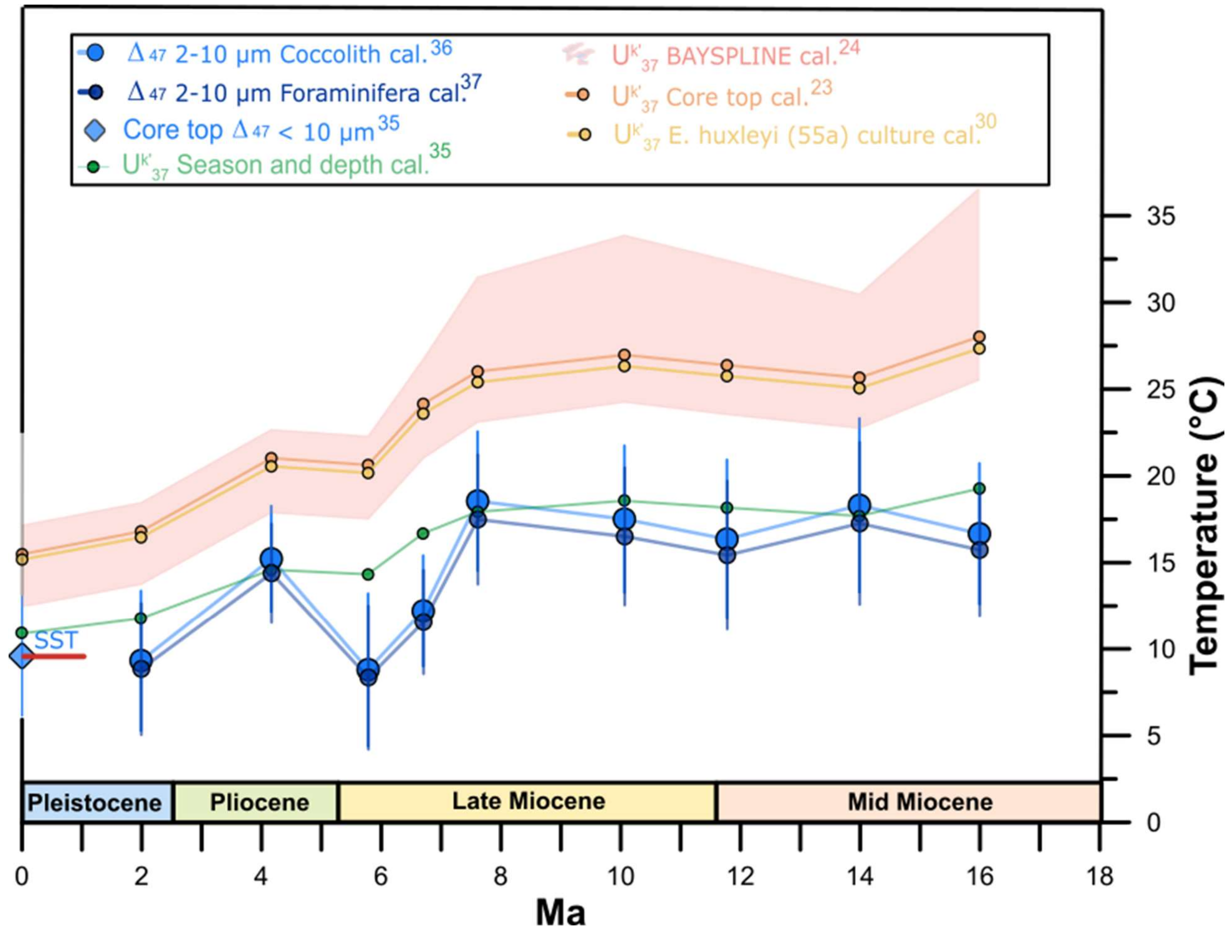


**Supplementary Figure 8. Coccolith clumped isotope temperature records obtained using different biogenic $\Delta_{47}$ calibrations, and alkenone temperature evolution in ODP Site 982.** $\Delta_{47}$ calcification temperatures from the pure coccolith 2-10 µm size fraction derived by applying the culture coccolith calibration ([36]; blue dots), and the foraminifera calibration ([37]; dark blue dots), showing the remarkable similarities between both records. Alkenone temperatures from the same samples calculated using the core top ([23]; orange dots), BAYSPLINE ([24]; pale pink shade), *E. huxleyi* 55a batch culture ([30]; pale yellow dots), and a calibration that considers the season and depth of production ([35]; green dots). Coretop alkenone and coccolith $\Delta_{47}$ temperatures from the study of Mejía et al. [35] in our same site are also included. Pale pink shaded area represents the 95% CI according to the BAYSPLINE calibration. Error bars in coccolith $\Delta_{47}$ calcification temperatures record denote the 95% CI.

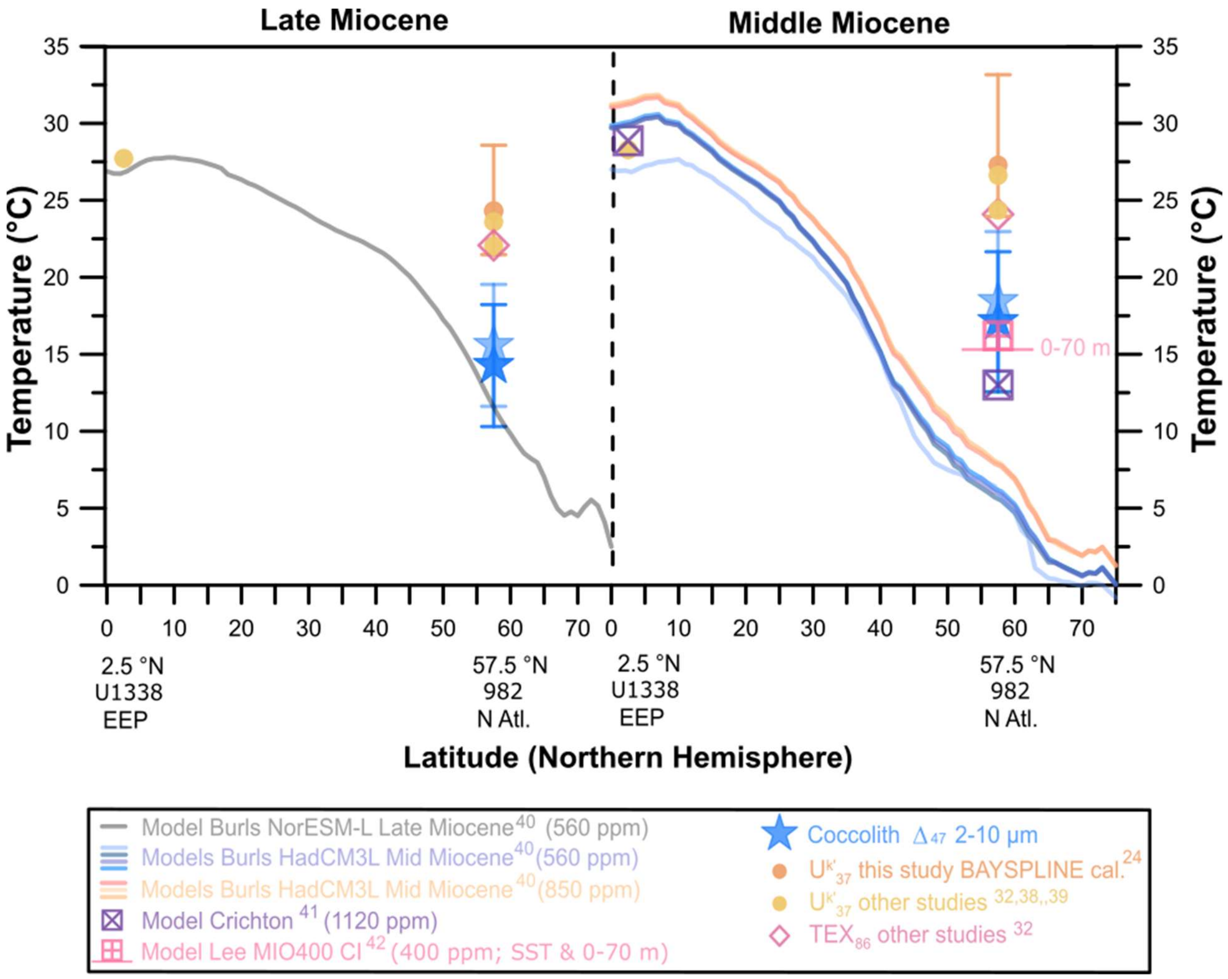


**Supplementary Figure 9. Late and Mid-Miocene latitudinal thermal gradient shown by coccolith clumped isotopes, alkenones and model simulations.** While alkenone temperatures calculated using widely-used calibrations suggest a small (Late Miocene: from 11.6 to 5.33 Ma) or negligible (Mid-Miocene: from last sample at 16 to 11.6 Ma) latitudinal thermal gradient ([32,38,39], and this study), coccolith clumped isotopes (as winter-spring at depth of production -dark blue star- and approximated surficial mean annual -pale blue star- temperatures) and Miocene model simulations[40–42] suggest a more modest polar amplification and a smaller flattening of the latitudinal thermal gradient. Modelled mean annual average 0-70 m water column temperatures from Lee et al.[42] shown as a horizontal pink line below mean annual SSTs for ODP Site 982, which differ by 0.9 °C. Errors for coccolith $\Delta_{47}$ calcification temperatures denote the 95% CI, and alkenone temperatures in this study denote the 95% CI according to the BAYSPLINE calibration.

**Supplementary Note 5. Coccolith $\Delta_{47}$ calcification temperatures comparison to modelled mean annual temperatures**

Traditionally, studies focused on model-data comparisons take directly proxy estimates and compare them to modelled data, without considering or correcting for potential biases of any kind. A typical case is when alkenone derived temperatures for the North Atlantic estimated using the BAYSPLINE alkenone calibration [24] are used for direct comparisons with modelled outputs, as the proxy data will be biased towards August-October temperatures (warm bias). Despite this, no efforts to "back-calculate" the seasonally-biased proxy to mean annual temperatures are traditionally conducted in these studies, nor are models comparing their seasonal outputs to proxies, but rather they use mean annual estimates directly.

Ideally, we would compare seasonally-modelled data at depth to our coccolith $\Delta_{47}$ calcification temperatures. However, due to the lack of publicly available winter-spring temperatures and the water column temperature structure from the majority of models[40], we have had to take the traditional approach and compare our coccolith $\Delta_{47}$ calcification temperatures directly to model mean annual temperatures. The only exception is the recently published model MIO400 CI which does include mean annual data of the water column for our study site[42] for the Mid-Miocene, allowing a more direct comparison to our coccolith $\Delta_{47}$ calcification temperatures. Although potential effects of seasonality cannot be isolated from the data of this model, both mean annual SSTs and mean annual 0-70 m (modern euphotic zone) temperatures match our coccolith $\Delta_{47}$ calcification temperatures with a difference of only 0.9 °C between depths.

In an attempt to account for potential seasonal and depth of production cold bias, we have as well back-calculated surface mean annual temperatures from our coccolith $\Delta_{47}$ calcification temperatures, assuming the difference between surface mean annual temperatures and winter-spring temperatures at production depth throughout the Miocene is similar to that of the modern ocean. We highlight that this assumption is likely not valid in a changing ocean since the Miocene, since we expect variability in for instance, the temperature contrast between seasons, coccolithophore season and depth of production depending on nutrient and mixing strength, and in the relationship between SSTs and temperatures at depth at our site.

To back-calculate the seasonal effect (~0.97 °C) we used temperatures from the season of production suggested by Behrenfeld et al. [20] (December-June) and compared them to mean annual SSTs for our site (10.57 °C). For the depth of production effect (~0.34 °C), we used the average SST for December-June (season of production [20]) and the average temperature of the water column between 0 and 70 m, considering this is a well-mixed site and that 71 m is the limit of the euphotic zone layer bellow which light during the season of production is not sufficient for photosynthesis. We calculate that assuming the ocean since the Miocene was as it is now, we could have a cold bias effect of 1.31 °C, which we have added to calculate surficial mean annual temperatures and compare them to modelled mean annual temperatures (Figure 5, Supplementary Figure 9).

It is worth noting that the comparatively larger maximum seasonal effect mentioned for the Holocene sample in the main text (up to 3 °C) is obtained when instead of using mean annual temperatures for calculations, SSTs of August to October (used in the BAYSPLINE calibration [24]; warmer than mean annual temperatures) are considered, and the season of production proposed by Broerse at al. [1] (March to May) is used instead of the broader December to June suggested by Behrenfeld et al. [20]. Regarding depth of production, for the Holocene sample maximum effect we used the maximum depth bias which is obtained for June for a 100 m depth (Supplementary Table 3; ~1.6 °C), instead of considering the average temperatures of the water column until the limit of the euphotic zone during months of production.

## References


1. Broerse, A. T. C., Ziveri, P., Van Hinte, J. E. & Honjo, S. Coccolithophore export production, species composition, and coccolith-CaCO3 fluxes in the NE Atlantic (34 °N 21 °W and 48 °N 21 °W). *Deep Sea Res 2 Top Stud Oceanogr* **47**, 1877–1905 (2000).
2. McIntyre, A. & Bé, A. W. H. Modern coccolithophoridae of the atlantic ocean-I. Placoliths and cyrtoliths. *Deep-Sea Research and Oceanographic Abstracts* **14**, 561–597 (1967).
3. Okada, H. & McIntyre, A. Seasonal distribution of modern coccolithophores in the western North Atlantic Ocean. *Mar Biol* **54**, 319–328 (1979).
4. Rosell-Melé, A., Comes, P., Müller, P. J. & Ziveri, P. Alkenone fluxes and anomalous UK'37 values during 1989-1990 in the Northeast Atlantic (48°N 21°W). *Mar Chem* **71**, 251–264 (2000).
5. Auderset, A. *et al.* Gulf Stream intensification after the early Pliocene shoaling of the Central American Seaway. *Earth Planet Sci Lett* **520**, 268–278 (2019).
6. Eiler, J. M. 'Clumped-isotope' geochemistry-The study of naturally-occurring, multiply-substituted isotopologues. *Earth Planet Sci Lett* **262**, 309–327 (2007).
7. Stolper, D. A., Eiler, J. M. & Higgins, J. A. Modeling the effects of diagenesis on carbonate clumped-isotope values in deep- and shallow-water settings. *Geochim Cosmochim Acta* **227**, 264–291 (2018).
8. Schrag, D. P., DePaolo, D. J. & Richter, F. M. Reconstructing past sea surface temperatures: Correcting for diagenesis of bulk marine carbonate. *Geochim Cosmochim Acta* **59**, 2265–2278 (1995).
9. Lear, C. H., Elderfield, H. & Wilson, P. A. Cenozoic deep-sea temperatures and global ice volumes from Mg/Ca in benthic foraminiferal calcite. *Science (1979)* **287**, 269–272 (2000).
10. Hassenkam, T., Johnsson, A., Bechgaard, K. & Stipp, S. L. S. Tracking single coccolith dissolution with picogram resolution and implications for CO2 sequestration and ocean acidification. *Proc Natl Acad Sci U S A* **108**, 8571–8576 (2011).
11. Jacob, D. E., Wirth, R., Agbaje, O. B. A., Branson, O. & Eggins, S. M. Planktic foraminifera form their shells via metastable carbonate phases. *Nat Commun* **8**, 1–9 (2017).
12. Stoll, H. *et al.* Insights on coccolith chemistry from a new ion probe method for analysis of individually picked coccoliths. *Geochemistry, Geophysics, Geosystems* **8**, Q06020 (2007).
13. Mejía, L. M. *et al.* Controls over $\delta^{44/40}$Ca and Sr/Ca variations in coccoliths: New perspectives from laboratory cultures and cellular models. *Earth Planet Sci Lett* **481**, (2018).

14. Chiu, T.-C. & Broecker, W. S. Toward better paleocarbonate ion reconstructions: New insights regarding the CaCO3 size index. *Paleoceanography* **23**, PA2216 (2008).

15. Mejía, L. M. *et al.* Effects of midlatitude westerlies on the paleoproductivity at the Agulhas Bank slope during the penultimate glacial cycle: Evidence from coccolith Sr/Ca ratios. *Paleoceanography* **29**, (2014).

16. Richter, F. M. & Liang, Y. The rate and consequences of Sr diagenesis in deep-sea carbonates. *Earth Planet Sci Lett* **117**, 553–565 (1993).

17. Hodell, D. A. *et al.* Anatomy of Heinrich Layer 1 and its role in the last deglaciation. *Paleoceanography* **32**, 284–303 (2017).

18. Young, J. R. & Ziveri, P. Calculation of coccolith volume and its use in calibration of carbonate flux estimates. *Deep Sea Res 2 Top Stud Oceanogr* **47**, 1679–1700 (2000).

19. Mignot, A., Ferrari, R. & Claustre, H. Floats with bio-optical sensors reveal what processes trigger the North Atlantic bloom. *Nat Commun* **9**, 1–9 (2018).

20. Behrenfeld, M. J., Doney, S. C., Lima, I., Boss, E. S. & Siegel, D. A. Annual cycles of ecological disturbance and recovery underlying the subarctic Atlantic spring plankton bloom. *Global Biogeochem Cycles* **27**, 526–540 (2013).

21. Beaufort, L., Couapel, M., Buchet, N., Claustre, H. & Goyet, C. Calcite production by coccolithophores in the south east Pacific Ocean. *Biogeosciences* **5**, 1101–1117 (2008).

22. Cortés, M. Y., Bollmann, J. & Thierstein, H. R. Coccolithophore ecology at the HOT station ALOHA, Hawaii. *Deep Sea Res 2 Top Stud Oceanogr* **48**, 1957–1981 (2001).

23. Müller, P. J., Kirst, G., Ruhland, G., Von Storch, I. & Rosell-Melé, A. Calibration of the alkenone paleotemperature index UK’37 based on core-tops from the eastern South Atlantic and the global ocean (60°N-60°S). *Geochim Cosmochim Acta* **62**, 1757–1772 (1998).

24. Tierney, J. E. & Tingley, M. P. BAYSPLINE: A New Calibration for the Alkenone Paleothermometer. *Paleoceanogr Paleoclimatol* **33**, 281–301 (2018).

25. Locarnini, R. A. *et al. World Ocean Atlas 2018, Volume 1: Temperature. World Ocean Atlas 2018* vol. 1 (NOAA Atlas NESDIS 81, 2018).

26. Sauzède, R. *et al.* Vertical distribution of chlorophyll a concentration and phytoplankton community composition from in situ fluorescence profiles: a first database for the global ocean. *Earth Syst Sci Data* **7**, 261–273 (2015).

27. Rosell-Melé, A. & Prahl, F. G. Seasonality of UK’37 temperature estimates as inferred from sediment trap data. *Quat Sci Rev* **72**, 128–136 (2013).

28. Filippova, A., Kienast, M., Frank, M. & Schneider, R. R. Alkenone paleothermometry in the North Atlantic: A review and synthesis of surface sediment data and calibrations. *Geochemistry, Geophysics, Geosystems* **17**, 1370–1382 (2016).

29. Newton, P. P., Lampitt, R. S., Jickells, T. D., King, P. & Boutle, C. Temporal and spatial variability of biogenic particles fluxes during the JGOFS northeast Atlantic process studies at 47°N, 20°W. *Deep-Sea Research Part I* **41**, 1617–1642 (1994).

30. Prahl, F. G., Muehlhausen, L. A. & Zahnle, D. L. Further evaluation of long-chain alkenones as indicators of paleoceanographic conditions. *Geochim Cosmochim Acta* **52**, 2303–2310 (1988).

31. D'Andrea, W. J., Theroux, S., Bradley, R. S. & Huang, X. Does phylogeny control U37K-temperature sensitivity Implications for lacustrine alkenone paleothermometry. *Geochim Cosmochim Acta* **175**, 168–180 (2016).

32. Super, J. R. *et al.* Miocene Evolution of North Atlantic Sea Surface Temperature. *Paleoceanogr Paleoclimatol* **35**, e2019PA003748 (2020).

33. Tierney, J. E. & Tingley, M. P. A TEX86 surface sediment database and extended Bayesian calibration. *Scientific Data 2015 2:1* **2**, 1–10 (2015).

34. Ho, S. L. & Laepple, T. Flat meridional temperature gradient in the early Eocene in the subsurface rather than surface ocean. *Nat Geosci* **9**, 606–610 (2016).

35. Mejía, L. M. *et al.* Clumped isotopes in globally distributed Holocene coccoliths reveal their habitat depth. *Earth Planet Sci Lett* **619**, 118313 (2023).

36. Clark, A. J., Torres-Romero, I., Jaggi, M., Bernasconi, S. M. & Stoll, H. M. Coccolithophorids precipitate carbonate in clumped isotope equilibrium with seawater. *Preprint egusphere 2023-2581* (2023) doi:10.5194/egusphere-2023-2581.

37. Meinicke, N., Reimi, M. A., Ravelo, A. C. & Meckler, A. N. Coupled Mg/Ca and Clumped Isotope Measurements Indicate Lack of Substantial Mixed Layer Cooling in the Western Pacific Warm Pool During the Last ~5 Million Years. *Paleoceanogr Paleoclimatol* **36**, e2020PA004115 (2021).

38. Herbert, T. D. *et al.* Late Miocene global cooling and the rise of modern ecosystems. *Nat Geosci* **9**, 843–847 (2016).

39. Rousselle, G., Beltran, C., Sicre, M. A., Raffi, I. & De Rafélis, M. Changes in sea-surface conditions in the Equatorial Pacific during the middle Miocene–Pliocene as inferred from coccolith geochemistry. *Earth Planet Sci Lett* **361**, 412–421 (2013).

40. Burls, N. J. *et al.* Simulating Miocene Warmth: Insights From an Opportunistic Multi-Model Ensemble (MioMIP1). *Paleoceanogr Paleoclimatol* **36**, e2020PA004054 (2021).

41. Crichton, K. A., Ridgwell, A., Lunt, D. J., Farnsworth, A. & Pearson, P. N. Data-constrained assessment of ocean circulation changes since the middle Miocene in an Earth system model. *Climate of the Past* **17**, 2223–2254 (2021).

42. Lee, D., Sarr, A. C., Acosta, R. P. & Poulsen, C. J. Multiple Ocean Equilibria and Decoupling of Miocene Atmospheric pCO2 and Regional Temperatures. *Paleoceanogr Paleoclimatol* **40**, e2025PA005126 (2025).
