# Supplementary material for: Coccolith clumped isotopes reveal modest rather than extreme northern high latitude amplification during the Miocene
Source: Nat Commun. 2025 Dec 9;16:10981. doi: 10.1038/s41467-025-65954-y (PMC12689775; doi:10.1038/s41467-025-65954-y)
Supplement: Supplementary file 2 — Description of Additional Supplementary Files [file 41467_2025_65954_MOESM2_ESM.pdf]

Description of Additional Supplementary Files

File name: Supplementary Dataset 1

Description: a. Sample details, including expedition, site, hole, core, type, section, and interval (cm). b. Complete clumped isotope raw dataset for all samples included in this study (sediment trap and downcore). c. Summary of clumped isotope and alkenone-derived temperatures, applying all relevant calibrations available in literature for both sediment trap and downcore samples.
